# Aggression is not blind: dominance and social history modulate murine responses to social instigation

**DOI:** 10.1007/s00213-025-06824-9

**Published:** 2025-06-04

**Authors:** Tomoya Nagai, Aki Takahashi

**Affiliations:** 1 Laboratory of Behavioral Neurobiology, University of Tsukuba, 1-1-1 Tennodai, Tsukuba, 411-8577 Ibaraki Japan; 2 Graduate School of Comprehensive Human Sciences, University of Tsukuba, Tsukuba, Ibaraki Japan; 3 Institute of Human Sciences, University of Tsukuba, Tsukuba, Ibaraki Japan

**Keywords:** Aggressive behavior, Escalated aggression, Social instigation, Social novelty, Social familiarity, Social dominance, Dominant-subordinate relationship, Mice

## Abstract

**Rationale:**

The social instigation procedure is a behavioral model used to induce escalated aggression in male mice. In this procedure, a brief indirect encounter with a novel rival male (instigator) placed in a tube enhances aggression toward an intruder in the subsequent agonistic encounter. However, social factors that drive this pro-aggressive effect remain unclear.

**Objectives:**

This study investigates how social novelty, familiarity, and dominant-subordinate hierarchy influence the pro-aggressive effect of social instigation.

**Methods:**

A male instigator in a perforated tube was placed in the test mouse’s home cage for 5 min, followed by the introduction of an intruder male to assess the test animal’s aggression. Different types of instigators were used to examine the roles of social novelty and dominance hierarchy. Aggressive behavior was compared to baseline aggression without social instigation.

**Results:**

Exposure to a novel male instigator escalated aggression, as indicated by shorter attack latency and increased frequency and duration of aggressive behaviors. In contrast, when a familiar intruder male was presented as the instigator, attack latency was reduced but the total amount of aggressive behaviors remained unchanged. When the instigator had an established dominant-subordinate relationship with the test mouse, aggressive behavior was not enhanced. However, when a familiar male without a prior dominant-subordinate relationship was used, aggressive behavior increased to levels comparable to those induced by a novel instigator.

**Conclusions:**

Social novelty and an ambiguous social hierarchy of the instigator promote the pro-aggressive effect of social instigation in male mice.

## Introduction

Aggressive behavior is conserved across a wide range of species, including mammals, fish and insects, aiming to harm conspecific rivals in order to acquire and protect resources such as territory, mate, and food (Miczek et al. 2001; Peake and Mcgregor 2004; Kravitz and Fernandez 2015). Aggressive behavior is directed not only at the primary source of provocation but also at an alternative target that was not the initial source. This phenomenon is known as displaced aggressive behavior in humans (Marcus-Newhall et al. 2000). Similarly, in various animal species from fish to rodents, prior brief exposure to a potential rival enhances aggressive behavior in subsequent aggressive encounter (Potegal 1992; Fish et al. 1999; Heiligenberg 1974; Kudryavtseva 1991). This facilitation of aggression is referred to as attack priming (Potegal and TenBrink 1984) or social instigation (Fish et al. 1999), processes that induce internal state of “aggressive arousal” (Potegal 1992) or “attack readiness” (Heiligenberg 1974), ultimately leading to the escalation of aggressive behavior.

The social instigation procedure for adult male mice involves placing a novel adult male (the instigator) inside a transparent tube with small holes and introducing it into the resident’s home cage. During this period, the resident receives visual, olfactory, and auditory cues from the instigator, perceiving it as an intruder in his territory but being unable to attack it directly. After the instigator is removed, a different intruder male is placed in the home cage, allowing the resident to engage in direct aggressive interactions. Studies have shown that the social instigation procedure shortens attack latency and increases the frequency of attack behaviors compared to aggression without prior instigation (Fish et al. 1999; de Almeida and Miczek 2002; Takahashi et al. 2015, 2022). Social instigation serves as a useful model for studying the neurobiological mechanisms of escalated aggression, as it can be implemented without genetic, pharmacological, or neurochemiacal manipulation (Miczek et al. 2013). Indeed, research using this model has implicated the serotonergic system (Fish et al. 1999; de Almeida and Miczek 2002), and specific brain areas, such as the medial amygdala and dorsal raphe nucleus, in mediating this aggression-facilitating effect (Potegal et al. 1996; Nordman et al. 2020; Takahashi et al. 2015, 2022).

However, a fundamental question remains: Why does the social instigation procedure enhance aggressive behavior? One potential explanation is frustration. During social instigation, the resident animal is unable to physically attack the instigator due to the protected tube, potentially inducing frustration by preventing the consummatory phase of aggressive behavior. Frustration is a well-established trigger for aggression, as proposed in the frustration-aggression hypothesis (Dollard et al. 1939). In animal models, exposure to unexpected non-reward in an operant conditioning context has been shown to increase aggressive behavior (de Almeida and Miczek 2002; Naik et al. 2024).

An alternative explanation is that the instigation-heightened aggression is a rational computational outcome. Animals adjust their level of aggressive behavior based on factors such as dominant-subordinate relationships (Holekamp and Strauss 2016), familiarity (Nakamura et al. 2007), and resource-holding potential (RHP) of their opponent (Maynard Smith and Parker 1976). If this is the case, the aggression facilitating effects of social instigation should vary depending on the characteristics of the stimulus presented as the instigator.

Prof. Klaus Miczek emphasized the importance of incorporating ethological perspectives into the study of the psychopharmacology of aggression. As he noted, “From an ethological viewpoint, a functional analysis of aggressive behavior has to consider the evolutionary forces of cohesion and dispersal that differentiate those species that are socially organized from those that live largely solitary lives,” and “Species-typical aggressive behavior and its functional significance in the survival of the individual and the species as a whole constitute the reference point for assessing maladaptive and excessive aggression” (Miczek 2001). In line with this view, this study aimed to examine the factors influencing social instigation-heightened aggression from multiple perspectives, including an ethological perspective. In Experiment 1, we examined the potential role of frustration caused by the inability to attack the instigator by comparing social instigation induced by a novel male and a familiar intruder male. In Experiment 2, we explored the effect of the instigator’s social characteristics, focusing on the dominant-subordinate relationship between the instigator and the resident male. In Experiment 3, we assessed the role of familiarity without prior aggressive interactions between the resident and the instigator male.

## Methods

### Animals

Male ICR/Crl (CD1) mice, aged 9 weeks, were purchased from Jackson Laboratory Japan and used as test animals (Experiment 1: 14 males, Experiment 2: 44 males, Experiment 3: 14 males). After being housed individually for at least one week in the animal facility in Building 2E, University of Tsukuba, they were used in the behavioral experiments. For intruders, male ICR/Jcl mice, aged 10 weeks or older, were purchased from Japan CLEA and used for the experiment after undergoing olfactory bulbectomy. Olfactory bulbectomy procedure was done to suppress aggressive behavior in the intruders (Denenberg et al. 1973). At least one week after the surgery, these mice were used in the experiment and were housed in groups of 3 or 4 animals. For instigators in the social instigation test, individually or group housed ICR male mice (obtained from either Jackson Laboratory or Japan CLEA), aged 10 weeks or older, were used as novel instigators. All behavioral experiments were conducted in the animal facility at Building 2E, University of Tsukuba. The animal rooms were maintained at 23 ± 2 °C with a 12-hour light/dark cycle (lights on at 12:00 pm, lights off at 12:00 am). During the experiments, all mice were housed in clear plastic cages (18 × 29 × 12 cm) with corn cob bedding, with water and food provided ad libitum. All experiments were performed under red light during the dark phase. All experimental procedures were approved and conducted in accordance with the Animal Care and Use Committee at the University of Tsukuba (approval number, 24–366).

### Aggressive behavior tests

#### Resident-Intruder test (RI test)

The RI test was conducted to observe the baseline territorial aggressive behavior of the test animals. Before the test, we replaced the cage lid with a clear acrylic panel. In the test, an intruder male was placed in the home cage of the test animal and the behavior of the test animal was observed and videotaped for 5 min. The first few days of RI testing were considered as habituation to aggressive encounters to establish a baseline for aggressive behavior. The same male intruder was presented to the resident during all trials, including both the RI test and the Inst test.

#### Social instigation (Inst) test

The Inst test was conducted to observe the increased aggressive behavior induced by social instigation. An instigator male, placed inside a transparent perforated tube (7 cm in diameter x 12 cm high, with multiple 5 mm diameter holes), was placed in a corner of the home cage of test animal and presented for 5 min. After the 5-minute period, the tube was removed and the intruder male (same animal used in the RI test) was introduced into the home cage, with behavior observed for 5 min. Behavior was videotaped for a total of 10 min, covering both the instigator presentation and the aggressive encounter with the intruder.

In this study, different types of instigators were presented; a novel animal (Novel-Inst test), the familiar intruder (Intruder-Inst test), a paired dominant/subordinate male (D/S-Inst test), and the partner mouse in the partition test (Partner-Inst test).

1. Novel-Inst test (Experiments 1–3)
  The instigator male, which was novel for the test animal was presented in the tube as the instigator for 5 min, followed by 5-minute RI test with the intruder. Different animal was presented as the instigator in the 1 st and 2nd Novel-Inst test.
2. Intruder-Inst test (Experiment 1)
  The intruder male, who has been used in prior RI trials, was presented in the tube as the instigator for 5 min, and this intruder was reintroduced into the home cage of test animal without the tube for 5 min. Since the test animal had already attacked this intruder in previous trials, the intruder was familiar and subordinate to the test animal.
3. D/S-Inst test (Experiment 2)
  The paired dominant or subordinate mouse encountered in the D/S test (described follow) was presented in a tube. If the test animal (resident) was dominant individual, the paired subordinate mouse was presented as the instigator, and if the resident was the subordinate animal, the paired dominant mouse was presented. After 5 min of the paired dominant or subordinate mouse presentation, 5-minute RI test with the intruder was conducted.
4. Partner-Inst test (Experiment 3)
  The partner mouse encountered in the partition test (described follow), in which familiar male but no prior fighting experience with the test animal, was presented as the instigator in the tube, followed by 5-minute RI test with the intruder

#### Dominant/Subordinate (D/S) test (Experiment 2)

The D/S test was conducted to establish a dominant-subordinate relationship between pairs of test animals. Each pair was predetermined and fixed throughout the experiment unless the animals failed to show aggressive behavior or to establish unilateral dominant-subordinate relationship. During the test, two animals were placed in a neutral cage for 10 min daily until a stable dominant/subordinate relationship was formed. Dominance was defined by one mouse consistently exhibiting unilateral aggressive behavior while the other ceased any aggressive act and showed submissive postures over three consecutive trials. If a unilateral dominance pattern was not observed within four trials, the combination of test animals was changed. Once the relationship was established, the D/S test was continued every other day to maintain the established relationship.

#### Partition test (Experiment 3)

A clear acrylic wall (28 × 10.5 cm) with multiple 6 mm holes was inserted in the middle of the home cage of the test animal. The test animal remained on one side of the partition, and an ICR male mouse (referred to as the partner mouse) was placed on the opposite side. The two mice were able to interact through the partition for 5 min. After the 5-minute interaction, the partner mouse and the partition were removed. The same partner mouse was used throughout all trials. This test was performed every other day, alternating with aggressive behavior tests (RI test and Inst test).

#### Behavior analysis

The behaviors of the test animals were analyzed by an observer using a free behavior analysis software (TanaMove0.07, created by A. Tanabe) based on recorded video files. The frequency and duration of four aggressive behaviors (attack bites, sideways threats, tail rattles and pursuits) and four non-aggressive behaviors (locomotion, rearing, self-grooming, and social contact, such as sniffing or allo-grooming directed at the intruder mice) were recorded. Frequency data was used for the analysis of attack bites, while duration data was used for all other indices. Additionally, the latency to the first attack bite (attack latency) and the total duration of all four aggressive behaviors (total aggressive behaviors) were calculated. The duration of sniffing directed toward the tube containing the instigator, locomotion, rearing and self-grooming were recorded during the presentation of the instigator.

#### Statistical analysis

The software GraphPad Prism 9.4.1 was used for statistical analysis. Since the Novel-Inst test and RI test were performed multiple times within the experiment, the mean values for each test were calculated and used in the analysis. In Experiments 1 and 3, a one-way repeated measures analysis of variance (ANOVA) with Geisser-Greenhouse correction (to account for deviations from sphericity) or a paired t test was performed. In Experiment 2, test animals were categorized into dominant and subordinate groups based on the D/S test, and a one-way repeated measures ANOVA or a paired t test were performed for each group. The Friedman test was used for analyzing attack latency across the experiments. If a significant main effect was found in a one-way repeated measures ANOVA or a Friedman test, *post hoc* multiple comparisons were conducted using either Tukey’s or Dunn's method (all two-sided).

Animals that did not show increased total aggressive behaviors during the Novel-Inst test compared to RI test were excluded from the analysis (Experiment 1: 3 males, Experiment 2: 6 dominant, 4 subordinate, Experiment 3: 2 males) because we aimed to focus on animals that showed instigation-heightened aggression using conventional social instigation method. In addition, some animals were excluded due to a reversal in the dominant-subordinate relationship during the test schedule, escaping from the tube during instigation, failure to record video, or absence of aggressive behavior across all trials (Experiment 2: 4 dominant, 5 subordinate).

## Results

### The effect of social instigation by a familiar intruder and a novel male (Experiment 1)

**Fig. 1:**
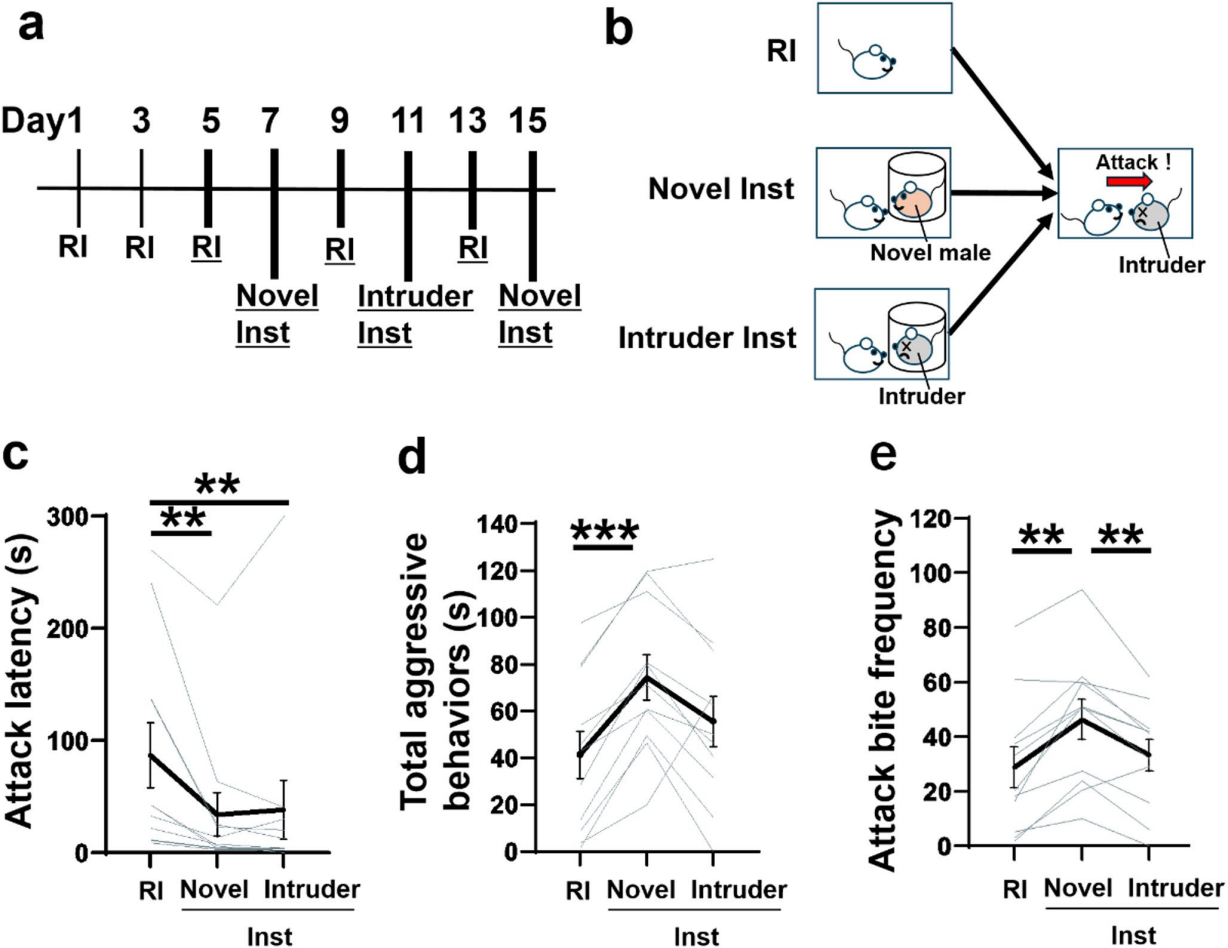
The effect of social instigation by a novel male and a familiar intruder male. (**A**) Timeline of Experiment 1. Two RI tests were initially conducted to habituate test mice to aggressive encounters with an intruder. Thereafter, RI tests and Inst tests were performed alternately every other day. In the Inst tests, two types of instigator were used: a novel male (Novel-Inst test) and the familiar intruder male (Intruder-Inst test). The Intruder-Inst test was conducted between two Novel-Inst tests. Throughout the experiment, the same intruder male was used for each test animal during aggressive encounters. The RI test, Novel-Inst test, and Intruder-Inst test on days 5–15 were used for statistical analysis (indicated by underlining). (**B**) Schematic of Experiment 1, in which either a novel male (Novel-Inst) or the intruder male (Intruder-Inst) was presented for 5 min as an instigator prior to the 5-min aggressive encounter. (**C**) Attack latency. A Friedman test revealed significant main effect of test conditions (Friedman statistic = 10.89, *p* = 0.0029), *n* = 9 biologically independent animals, with *post hoc* Dunn’s multiple comparison tests (two-sided). (**D**) The duration of total aggressive behavior. The main effect of test conditions was significant in one-way repeated measures ANOVA (*F*(1.482, 14.82) = 12.12, *p* = 0.0015), *n* = 11 biologically independent animals, with *post hoc* t-test using Tukey’s multiple comparison tests (two-sided). (**E**) The frequency of attack bites. One-way repeated measures ANOVA showed significant main effect of test conditions (F(1.827, 18.27) = 12.58, *p* = 0.0005), *n* = 11 biologically independent animals, with *post hoc* t-test using Tukey’s multiple comparison tests (two-sided). Black lines represent mean values ± SEM, and gray lines indicate each individual’s data. *p < 0.05, **p < 0.01, ***p < 0.001

We first compared the effects of social instigation on aggressive behavior by presenting either a familiar intruder or a novel male as the instigator. Before the social instigation tests, each resident male mouse had encountered the same intruder male at least three times in the RI tests, gaining repeated winning experiences against this intruder (Fig. 1A). In the social instigation test, we presented either a novel male (Novel-Inst) or the familiar intruder (Intruder-Inst) for 5 min. Then, an intruder male was placed directly into the homecage to observe direct aggressive interactions for 5 min (Fig. 1B). We hypothesized that if frustration induce by the social instigation procedure escalates aggression, then the presentation of the familiar intruder would facilitate aggressive behavior similarly to or more than instigation by a novel male (Fig. 1B), due to the inability to attack an opponent that is usually attackable.

During the 5-minute presentation of the instigator, we measured the duration of sniffing toward the tube containing the instigator as an index of social interest. Test animals spend slightly more time sniffing the novel male (Novel-Inst) compared to the familiar intruder (Intruder-Inst), but this difference was not significant (Table 1). Other behavioral indices, such as the duration of locomotion, rearing, and self-grooming, showed no differences during the social instigation period between the Novel-Inst and Intruder-Inst (Table 1).

**Table 1:** Behaviors during 5-min social instigation and 5-min aggressive encounter in experiment 1 (Intruder-Instigation test)

	RI	Novel-Inst	Intruder-Inst
Behaviors during social instigation			
Sniffing toward the tube (s)	-	111.7 ± 10.6	91.5 ± 11.0
Locomotion (s)	-	57.0 ± 4.1	55.7 ± 4.4
Rearing (s)	-	106.9 ± 11.0	122.6 ± 13.0
Self-grooming (s)	-	6.3 ± 0.7	6.5 ± 0.8
Behaviors during aggressive encounter			
*Aggressive behaviors*			
Attack bites (frequency)	28.8 ± 7.5	46.4 ± 7.2**	33.4 ± 5.8††
Tail rattles (s)	26.7 ± 6.4	56.0 ± 7.4***	40.5 ± 7.9* †
Sideways threats (s)	30.6 ± 8.2	55.1 ± 7.8***	45.1 ± 9.6
Pursuits (s)	1.6 ± 0.5	3.3 ± 0.8*	1.3 ± 0.3†
*Non-aggressive behaviors*			
Locomotion (s)	124.3 ± 22.1	109.5 ± 14.2	66.7 ± 6.8†
Self-grooming (s)	5.8 ± 1.3	7.8 ± 1.4	6.2 ± 1.7
Rearing (s)	57.1 ± 6.2	45.9 ± 4.2	40.3 ± 8.1*
Social contact (s)	71.0 ± 17.6	58.0 ± 16.7	65.1 ± 22.7

Values are mean ± SEM. *P < 0.05, ** *P* < 0.01, ***P < 0.001 vs. RI, †*P* < 0.05, ††*P* < 0.01 between Novel-Inst vs. Intruder-Inst

During the aggressive encounter, both Novel-Inst and Intruder-Inst tests significantly shortened the time to the first attack compared to the RI test without social instigation (Fig. 1C). On the other hand, the duration of total aggressive behaviors was significantly increased only in the Novel-Inst test, not in the Intruder-Inst test, compared to the RI test (Fig. 1D). A detailed behavioral analysis further showed that aggressive components of behaviors increased only in the Novel-Inst test and not in the Intruder-Inst test (Fig. 1E; Table 1). *Post hoc* analysis revealed significant differences between Novel-Inst test and Intruder-Inst test in the frequency of attack bites, as well as the durations of tail rattles and pursuits (Fig. 1E; Table 1). For non-aggressive behaviors, the durations of locomotion and rearing were significantly lower in the Intruder-Inst test compared to Novel-Inst test, whereas no significant differences were observed between tests for self-grooming and social contact (Table 1).

These results suggest that social instigation by the familiar intruder mice, which did not represent a novel threat, rather had an inhibitory effect on the pro-aggressive effect of social instigation, with the exception of attack latency.

### The effect of social instigation by dominant and subordinate mice (Experiment 2)

**Fig. 2:**
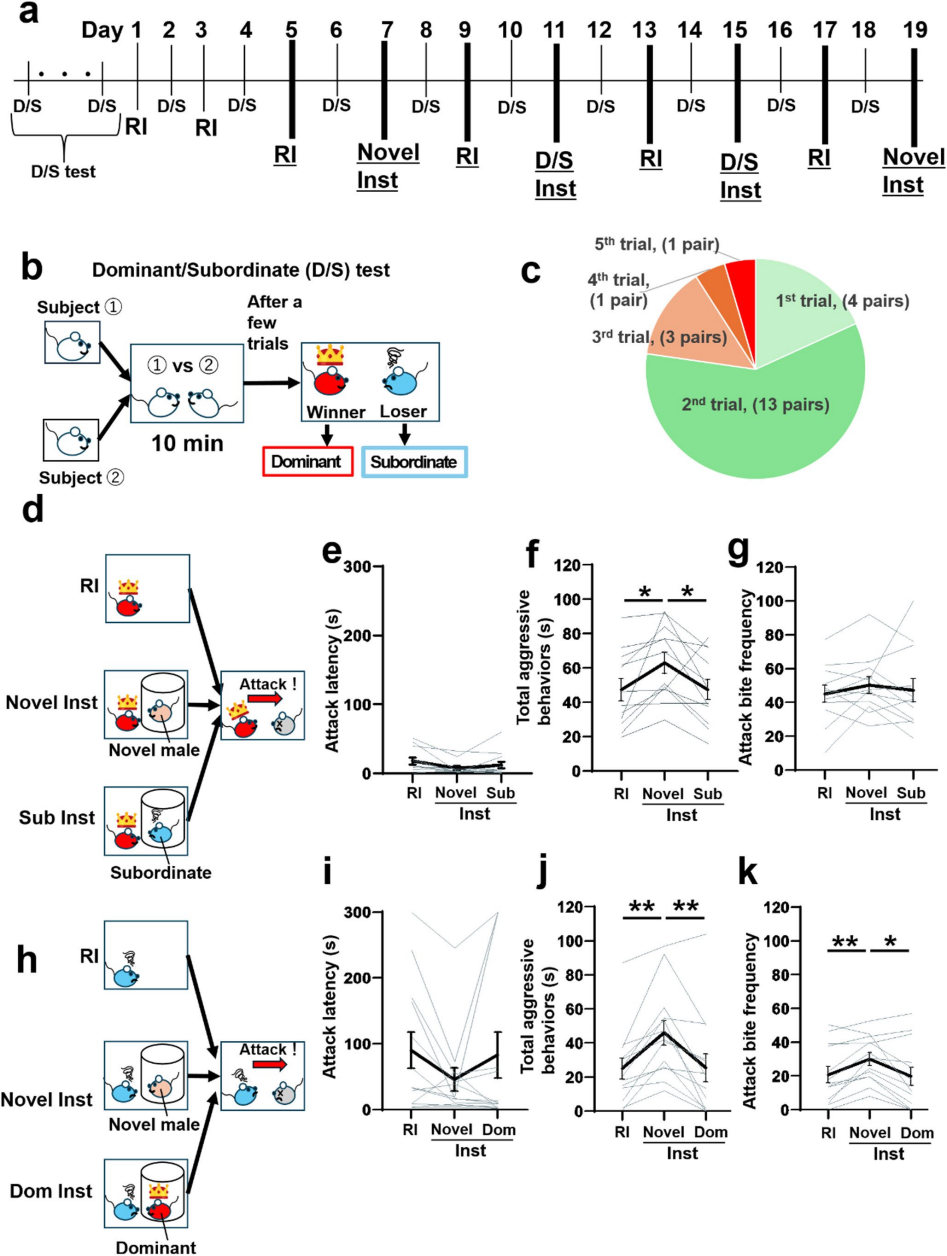
The effect of social instigation by a dominant or subordinate male. (**A**) Timeline of Experiment 2. Dominant/Subordinate (D/S) tests were conducted daily until a stable dominant-subordinate relationship was established. Once established, D/S tests were conducted every other day, alternating with aggression tests (RI tests and Inst tests) to maintain the relationship. After at least two days of RI tests for habituation to aggressive encounters, RI tests and Inst tests were performed alternately every other day. In the Inst tests, two types of instigator were used: a novel animal (Novel-Inst test) and the paired dominant or subordinate mouse from the D/S tests (D/S-Inst test). For the D/S-Inst tests (Day 11 and Day 15), one mouse from each D/S pair served as the instigator while the other was tested as the resident. Each test animal alternated between being the resident and instigator, with the order counterbalanced across individuals. Aggression tests (RI tests, Novel-Inst test, and D/S-Inst tests) analyzed for statistical comparisons are underlined. (**B**) Schematic of the D/S test. A predetermined pair of test animals was placed in a novel home cage and allowed to interact for 10 min to establish a dominant-subordinate relationship. (**C**) Number of trials required for each pair to show one-directional attack behavior (only from the dominant to subordinate) in the D/S test. (**D**) Schematic of the RI test, Novel-Inst test, and Sub-Inst test in dominant mice in the D/S test. Either a novel male (Novel-Inst) or the paired subordinate male (Sub-Inst) was presented for 5 min as an instigator before the 5-minute aggressive encounter. (**E**) Attack latency. A Friedman test revealed no significant main effect of test conditions. (**F**) Duration of total aggressive behavior. A one-way repeated measures ANOVA showed a significant main effect of test conditions (*F*(1.916, 21.08) = 6.316, *p* = 0.0077, *n* = 12 biologically independent animals, with *post hoc* t test using Tukey’s multiple comparison tests (two-sided)). (**G**) Frequency of attack bites. A one-way repeated measures ANOVA revealed no significant main effect of test conditions, *n* = 12 biologically independent animals. (**H**) Schematic of the RI test, Novel-Inst test, and Dom-Inst test in subordinate mice in the D/S test. A novel male (Novel-Inst) or paired dominant male (Dom-Inst) was presented as an instigator prior to aggressive encounter. (**I**) Attack latency. A Friedman test revealed no main effect of test conditions. (**J**) Duration of total aggressive behavior. A one-way repeated measures ANOVA showed significant main effect of test conditions (*F*(1.909, 22.91) = 12.12, *p* = 0.0003, *n* = 13 biologically independent animals, with *post hoc* t test using Tukey’s multiple comparison test (two-sided)). (**K**) Frequency of attack bites. A one-way repeated measures ANOVA showed a significant main effect of test conditions (F(1.382, 16.58) = 5.930, *p* = 0.0186), *n* = 13 biologically independent animals, *post hoc* t test with Tukey’s multiple comparison tests (two-sided). Black lines represent mean values ± SEM, and gray lines indicate each individual data points. *p < 0.05, **p < 0.01

Given that the pro-aggressive effect of the social instigation is modulated by the characteristics of the instigator, as shown in Experiment 1, we next examined the effect of dominant-subordinate relationship between the test animal and the instigator on aggressive behavior. Before conducting social instigation tests, we performed a dominant/subordinate (D/S) test, in which two test mice encountered each other in a novel cage for 10 min. This test was repeated until a stable dominant-subordinate relationship was established (Fig. 2A, B). Approximately 77% of pairs established dominance within two trials, but one pair required five D/S encounters to establishing a dominant-subordinate relationship (Fig. 2C). Once one-directional attacks were observed, we continued the D/S tests to confirm a stable relationship for at least three consecutive trials.

In the social instigation tests, we presented either a novel male (Novel-Inst) or the opponent from the D/S test (D/S-Inst). For the dominant test males, the presented D/S pair animal was a subordinate, and vice versa. Therefore, we separately analyzed the results for dominant test animals presented with a subordinate instigator (Sub-Inst test; Fig. 2D) and for subordinate test animals presented with a dominant instigator (Dom-Inst test; Fig. 2H) to examine their aggressive behavior toward an intruder male.

#### Presentation of subordinate male as instigator (Sub-Inst) in dominant test animals

During the social instigation, no significant differences were found between the Novel-Inst and Sub-Inst tests in the duration of the sniffing toward the instigator, locomotion, or rearing, except for self-grooming, which was significantly longer in the Sub-Inst test compared to Novel-Inst test (Table 2).

**Table 2:** Behaviors during 5-min social instigation and 5-min aggressive encounter of dominant test animals in experiment 2 (Subordinate-Instigation test)

	RI	Novel-Inst	Sub-Inst
Behaviors during social instigation			
Sniffing toward the tube (s)	-	110.7 ± 7.8	106.6 ± 6.2
Locomotion (s)	-	62.3 ± 3.7	61.3 ± 4.1
Rearing (s)	-	127.9 ± 7.4	131.3 ± 5.5
Self-grooming (s)	-	8.1 ± 1.1	10.3 ± 1.5**†**
Behaviors during aggressive encounter			
*Aggressive behaviors*			
Attack bites (frequency)	45.2 ± 5.0	50.2 ± 4.9	47.3 ± 6.9
Tail rattles (s)	40.6 ± 6.4	55.6 ± 6.6*	39.8 ± 5.4**†**
Sideways threats (s)	34.4 ± 6.3	44.2 ± 6.2*	34.3 ± 5.7
Pursuits (s)	1.5 ± 0.7	1.6 ± 0.6	1.9 ± 0.9
*Non-aggressive behaviors*			
Locomotion (s)	82.8 ± 3.7	92.1 ± 3.2	79.4 ± 8.5
Self-grooming (s)	12.1 ± 1.1	16.1 ± 2.0	11.6 ± 1.8
Rearing (s)	75.8 ± 6.3	68.0 ± 6.4	68.6 ± 9.4
Social contact (s)	73.9 ± 13.9	49.0 ± 8.5	66.9 ± 15.0

Values are mean ± SEM. *P < 0.05 vs. RI, †*P* < 0.05 between Novel-Inst.vs Sub-Inst

During the aggressive encounter with the intruder, no significant differences in attack latency was observed (Fig. 2E). However, the duration of total aggressive behaviors was significantly longer in the Novel-Inst test, but not in the Sub-Inst test, compared to the RI test, with a significant difference between Novel-Inst and Sub-Inst tests (Fig. 2F). A detailed behavioral analysis indicated that some aggressive components of behaviors, such as the durations of sideways threats and tail rattles, significantly increased in the Novel-Inst test but not in the Sub-Inst test compared to the RI test. However, no significant main effect of test conditions was observed in the frequency of attack bites and the duration of pursuits (Fig. 2G; Table 2). No significant differences between tests were found for non-aggressive behaviors (Table 2).

#### Presentation of dominant male as instigatior (Dom-Inst) in subordinate test animals

During the social instigation, no significant differences were found between these tests in any behavioral indices including the duration of the sniffing toward the instigator (Table 3). During the aggressive encounter with the intruder, all subordinate test animals in the D/S test exhibited aggressive behavior toward non-aggressive, olfactory bulbectomized intruder male, although the level of aggression was lower than those of D/S dominant males (Fig. 2I-K). Again, no significant differences in attack latency were observed across tests (Fig. 2I). However, the duration of total aggressive behaviors was significantly increased in the Novel-Inst test but not in the Dom-Inst test compared to the RI test (Fig. 2J). Consistently, detailed behavioral analysis showed that aggressive components of behaviors increased in the Novel-Inst test but not in the Dom-Inst test compared to the RI test. Significant differences were found between the Novel-Inst and the Dom-Inst tests in the frequency of attack bites and the durations of tail rattles and sideways threats (Fig. 2K; Table 3). For non-aggressive behaviors, the Novel-Inst test, but not the Dom-Inst test, showed longer duration of locomotion compared to the RI test. Meanwhile, only the Dom-Inst test showed the decrease in duration of rearing and an increase in durations of self-grooming and social contact compared to the RI test (Table 3).

**Table 3:** Behaviors during 5-min social instigation, and 5-min aggressive encounter of subordinate test animals in experiment 2 (Dominant-Instigation test)

	RI	Novel-Inst	Dom-Inst
Behaviors during social instigation			
Sniffing toward the tube (s)	-	113.3 ± 9.0	101.7 ± 7.9
Locomotion (s)	-	61.6 ± 2.9	56.7 ± 2.7
Rearing (s)	-	116.1 ± 6.6	120.0 ± 8.4
Self-grooming (s)	-	6.2 ± 1.0	4.9 ± 2.1
Behaviors during aggressive encounter			
*Aggressive behaviors*			
Attack bites (frequency)	20.8 ± 4.8	30.1 ± 3.8**	19.4 ± 5.4**†**
Tail rattles (s)	22.2 ± 6.0	42.5 ± 7.3**	23.4 ± 8.1**††**
Sideways threats (s)	14.7 ± 3.6	27.6 ± 4.7**	15.3 ± 4.8**††**
Pursuits (s)	0.5 ± 0.2	1.2 ± 0.4	0.7 ± 0.2
*Non-aggressive behaviors*			
Locomotion (s)	78.2 ± 6.6	95.1 ± 6.4***	74.3 ± 7.6**†**
Self-grooming (s)	6.8 ± 1.2	9.3 ± 1.7	10.9 ± 1.6**
Rearing (s)	62.0 ± 8.5	58.1 ± 7.7	48.4 ± 7.9*
Social contact (s)	118.8 ± 13.6	105.6 ± 13.7	139.5 ± 14.7**

Values are mean ± SEM. **P < 0.01, ***P < 0.001 vs. RI, †*P* < 0.05, ††*P* < 0.01 Novel-Inst vs. Dom-Inst

These results indicate that the attenuation of the pro-aggressive effect of social instigation occurs not only when the instigator is subordinate but also when it is dominant relative to the test animal, suggesting that social familiarity may influence instigation-heightened aggression.

## The effect of the social instigation by familiar partner mice (Experiment 3)

**Fig. 3:**
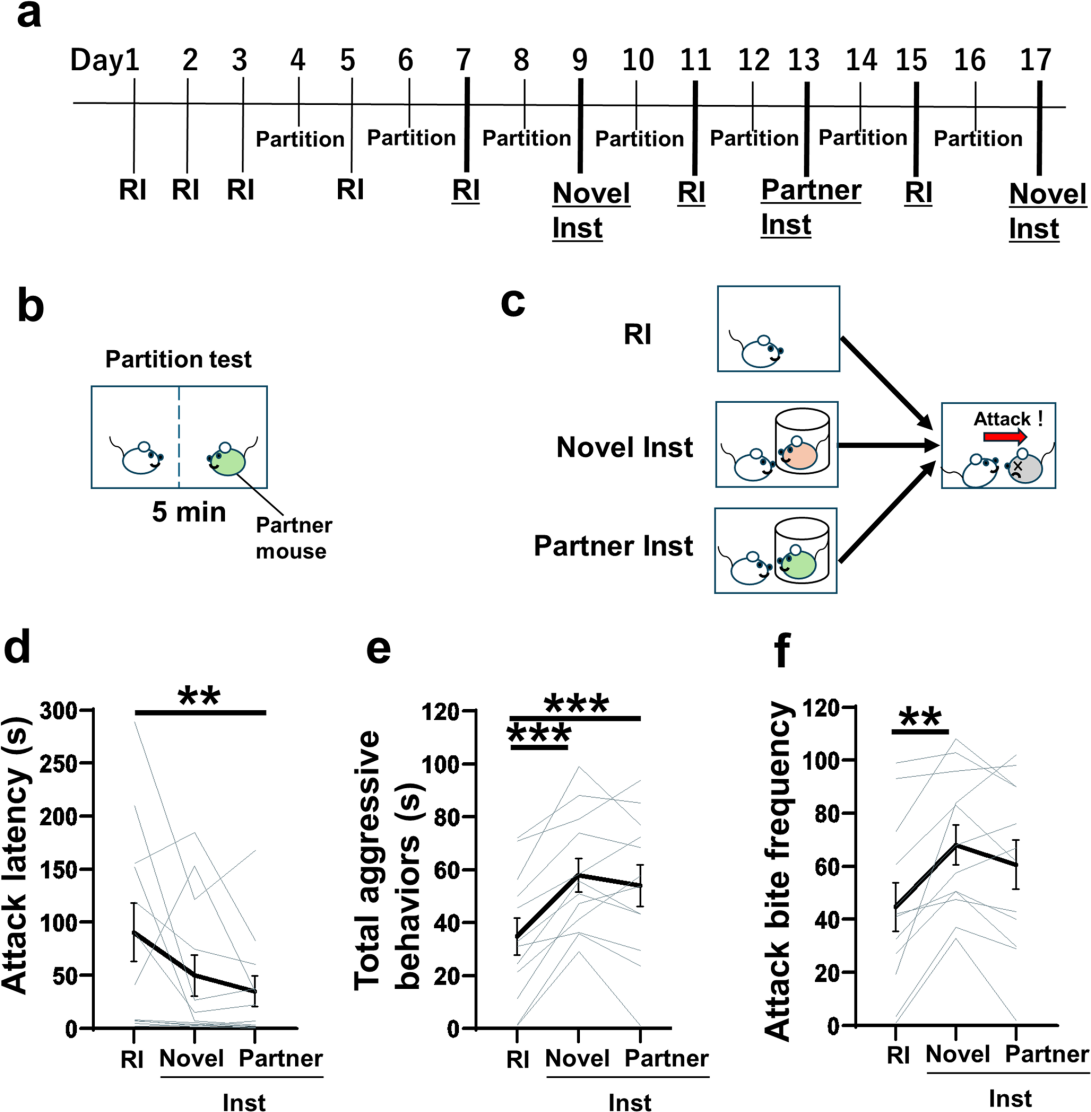
The effect of social instigation by familiar partner mice. (**A**) Timeline of Experiment 3. Four RI tests were conducted to habituate test mice to aggressive encounter. Two Partition tests were performed before the experiment to establish social familiarity. Subsequently, RI tests, the Novel-Inst tests, and a Partner-Inst test (the social instigation by a partner mouse) were conducted. Partition tests were performed every other day between aggression tests to maintain social familiarity. The aggression tests (RI test, Novel-Inst test, and Partner-Inst test) analyzed for statistical comparisons are underlined. (**B**) Partition test. The test animal interacted with a partner mouse across the partition for 5 minutes in the home cage of the test animal. (**C**) Schematic of the RI test, Novel-Inst test, and Partner-Inst test. Either a novel male (Novel-Inst) or a familiar partner male (Partner-Inst) was presented as an instigator before RI test. (**D**) Attack latency. A Friedman test revealed a significant main effect of test conditions (Friedman statistic = 12.17, *p* = 0.0023), *n* = 12 biologically independent animals, with *post hoc* Dunn’s multiple comparison test (two-sided). (**E**) Duration of total aggressive behavior. A one-way repeated measures ANOVA showed significant main effect of test conditions (*F*(1.895, 20.84) = 21.62, *p* < 0.0001), *n* = 12 biologically independent animals, with *post hoc* t test using Tukey’s multiple comparison tests (two-sided)). (**F**) Frequency of attack bites. A one-way repeated measures ANOVA showed a significant main effect of test conditions (*F*(1.345, 14.80) = 8.788, *p* = 0.0062), *n* = 12 biologically independent animals, with *post hoc* t test using Tukey’s multiple comparison tests (two-sided). Black lines represent mean values ± SEM, and gray lines indicate each individual’s data. *p < 0.05, **p < 0.01, ***p < 0.001

To examine the possible involvement of social familiarity in social instigation-heightened aggression, we conducted a social instigation test with a familiar partner that did not have a dominant-subordinate relationship with the test animal. Before social instigation tests, we conducted a partition test, in which the test animal was exposed to a male mouse (partner) through a perforated divider in the home cage for five minutes. This test was repeated with the same partner throughout the experiment to maintain social familiarity (Fig. 3A, B). In the social instigation test, we presented either a novel male (Novel-Inst) or the familiar partner male from the partition test (Partner-Inst) and examined aggressive behavior toward an intruder male (Fig. 3C).

During social instigation, the duration of sniffing toward the instigator was longer, and the duration of self-grooming was shorter, in the Novel-Inst test compared to the Partner-Inst test, suggesting that subjects detected differences in social familiarity (Table 4). In the aggressive encounter with intruder, the Partner-Inst test significantly shortened the attack latency compared to the RI test (Fig. 3D). In addition, both the Partner-Inst test and the Novel-Inst test showed significant increase in the duration of total aggressive behaviors compared to the RI test (Fig. 3E). Indeed, most aggressive components of behaviors increased in both the Novel-Inst and the Partner-Inst tests compared to the RI test (Fig. 3F; Table 4). Among non-aggressive behaviors, the duration of locomotion was significantly increased in the Partner-Inst test compared to both the RI test and the Novel-Inst test, whereas no significant differences between tests were observed in other non-aggressive behaviors (Table 4).

**Table 4:** Behaviors during 5-min social instigation, and 5-min aggressive encounter in experiment 4 (Partner-Instigation test)

	RI	Novel-Inst	Partner-Inst
Behaviors during social instigation			
Sniffing toward the tube(s)	-	136.7 ± 11.8	113.9 ± 9.3**†**
Locomotion (s)	-	49.8 ± 2.4	48.1 ± 3.3
Rearing (s)	-	106.5 ± 9.3	104.8 ± 9.8
Self-grooming (s)	-	8.7 ± 1.0	6.0 ± 0.7**†**
Behaviors during aggressive encounter			
*Aggressive behaviors*			
Attack bites (frequency)	44.8 ± 9.2	68.2 ± 7.5***	60.8 ± 9.2*
Tail rattles (s)	29.6 ± 5.7	51.8 ± 6.0***	48.2 ± 7.1***
Sideways threats (s)	16.9 ± 4.0	26.4 ± 3.7**	26.2 ± 5.2**
Pursuits (s)	0.9 ± 0.4	1.5 ± 0.4	1.9 ± 0.8
*Non-aggressive behaviors*			
Locomotion (s)	79.7 ± 3.6	80.3 ± 4.8	98.3 ± 6.7* †
Self-grooming (s)	6.2 ± 1.2	7.7 ± 1.5	6.4 ± 1.4
Rearing (s)	76.3 ± 9.0	70.8 ± 11.2	88.3 ± 14.0
Social contact (s)	82.1 ± 11.1	62.4 ± 10.8	87.7 ± 16.8

Values are mean ± SEM. *P < 0.05, **P < 0.01, ***P < 0.001 vs. RI, †*P* < 0.05 between Novel-Inst vs. Partner-Inst

These results indicate that mere familiarity of the instigator does not inhibit the pro-aggressive effect of social instigation.

## Discussion

In this study, we focused on the characteristics of the instigator animal to examine factors that influence social instigation-heightened aggression in male mice. Consistent with previous studies, pre-exposure to a novel male rival caused escalation of aggressive behavior, including shorter attack latency and increased frequencies and durations of aggressive behaviors in the subsequent agonistic encounter (Fish et al. 1999; de Almeida and Miczek 2002; Takahashi et al. 2015, 2022). However, when a familiar intruder male was presented as the instigator, we observed only a reduction in attack latency, with no changes in the overall amount of aggressive behaviors. In addition, when the instigator was an animal with already established dominant-subordinate relationship to the test animal, aggressive behavior did not increase. In contrast, when a familiar male without a prior dominant-subordinate relationship was presented, the test mice showed strong increases in aggressive behaviors comparable to those induced by a novel instigator. These findings suggest that an established dominant-subordinate relationship, rather than mere familiarity, between the test animal and the instigator attenuates the pro-aggressive effect of social instigation.

Our original hypothesis considered the possible involvement of frustration in aggression escalation by the social instigation procedure. Frustration induced by the omission of an expected reward has been shown to increase the frequency and duration of operant responses (e.g. lever pressing), alter running speed, and enhance aggressive behavior (Amsel and Roussel 1952; Azrin et al. 1966; de Almeida and Miczek 2002; Hull 1952; Naik et al. 2024; Vasquez et al. 2021). Increased aggression due to frustration has been observed across various species, including rodents, birds, and humans (Caprara 1982; Dollard et al. 1939; Cherek and Pickens 1970; Thompson and Bloom 1966). During the social instigation procedure, the test animals are unable to directly attack the instigator male due to the protective tube, which may induce a state of frustration. Previous studies have shown that the expressing aggressive behavior is rewarding for male mice (Golden et al. 2019). In this study, we used the same intruder male for each test animal throughout the experiments, and we hypothesized that presenting a familiar intruder as an instigator would trigger a stronger motivation to express the consummatory phase of aggression, leading to greater frustration compared to a novel opponent. However, the results from Experiment 1 indicated that social instigation with a familiar intruder did not increase the duration and frequency of aggressive behavior, whereas a novel instigator did. This suggests that the level of social novelty actively modulates social instigation-heightened aggression. On the other hand, we observed similar reductions in the attack latency following social instigation with both the familiar intruder and the novel male, compared to the RI test without social instigation. These results suggest that the social instigation procedure facilitates the initiation of aggressive behavior (i.e. readiness to attack) regardless of the social characteristics of instigator, and that frustration may be involved in this effect.

In the Intruder-Inst test of Experiment 1, the intruder mice were not only familiar subordinates but also the same animals presented in the subsequent agonistic encounter, whereas in the Novel-Inst test, the instigator and the intruder were different animals. To control for this procedural difference and to examine the effect of the dominant-subordinate relationship between the instigator and the test animal on the pro-aggressive effect of social instigation, we next established dominant-subordinate relationships between pairs of test animals and presented each as an instigator. Consistent with the findings from the intruder-Inst test, instigation by subordinate D/S pair mice inhibited the pro-aggressive effect of social instigation in dominant test animals. Previous studies have shown that in the RI test, resident males exhibited less aggressive behavior toward subordinate males than toward novel males (Parmigiani and Brain 1983), suggesting that subordinate males are less potent stimuli for eliciting aggressive behavior. Interestingly, in our study, subordinate test males in the D/S test also displayed aggressive behavior toward the intruder male in the RI test, and social instigation with a novel male facilitated aggressive behavior compared to baseline RI aggression. Notably, in this study we excluded animals that did not show instigation-heightened aggression in the Novel-Inst test, as individual differences exist in the pro-aggressive effect of social instigation (Fish et al. 1999). However, the proportion of excluded subjects was nearly the same between dominant and subordinate groups, suggesting that D/S subordinate males can show instigation-heightened aggression similar to dominant males. Nevertheless, when dominant D/S pair mice were presented as instigators, the pro-aggressive effect of social instigation was inhibited. Previous studies have reported that resident male mice never show aggressive behavior when dominant males are presented as opponents (Wei et al. 2023). In contrast, in this study, even when dominant mice were presented as instigators in the Dom-Inst test, the test animals showed similar levels of aggression toward subordinate intruder males as observed in the RI test. Taken together, these results suggest that presenting either dominant or subordinate mouse as an instigator does not enhance aggression, indicating that a pre-established dominant-subordinate relationship between the test animal and the instigator plays a modulatory role in social instigation-heightened aggression.

Our results further suggest that the mere social familiarity does not influence the pro-aggressive effect of social instigation. When familiar partner mice without an established dominant-subordinate relationship were presented as instigators, test animals exhibited increased aggressive behavior comparable to that observed during social instigation with a novel male. Thus, the presence of an established dominant-subordinate relationship, rather than familiarity alone, appears to be an important modulating factor in social instigation-heightened aggression. In the field of ethology, a phenomenon known as the ‘dear enemy effect’ has been observed (Fisher 1954). In several animal species living in the wild, individuals in a territory exhibit ‘relatively stronger defensive responses to territorial intrusion by strangers rather than neighbors’ (Christensen and Radford 2018). In this context, “neighbors” refer to familiar individuals encountered around the borders of the territory. The findings of the present study could be interpreted through the lens of the dear enemy effect. In Experiment 3, the effect of social instigation by partner mice was similar to that observed with novel male mice, despite the fact that partner mice had previously intruded into the territory of the test animals during the partition test and could be considered “neighbors”. This suggests that the dear enemy effect in social instigation may require the establishment of a dominant-subordinate relationship, which partner mice lack.

Locomotor activity is considered an indicator of behavioral arousal (Valentinuzzi et al. 2000), with overlapping yet distinct neural mechanisms involved (Lu et al. 2020; Vinck et al. 2015), and heightened arousal has been linked to increased aggression (Jaffe et al. 1974; Zillmann et al. 1974). In Experiment 1, during aggressive encounter, locomotion duration was longer in the Novel-Inst test than in the Intruder-Inst test. Similarly, in Experiment 2, subordinate subjects showed longer locomotion duration in the Novel-Inst test compared to both the RI and Dom-Inst tests, while dominant animals showed a similar trend, though it did not reach statistical significance. These results suggest that arousal levels induced by a familiar instigator with a dominant-subordinate relationship were lower than those induced by a novel instigator, potentially contributing to the reduced pro-aggressive effect. By contrast, In Experiment 3, locomotion duration was longer in the Partner-Inst test than in the other tests, suggesting that a familiar instigator without an established dominant-subordinate relationship still induces arousal, possibly even more than a novel instigator male.

Although both Dom-Inst and Sub-Inst tests induced similar changes in aggressive behaviors and locomotion, differences emerged in other non-aggressive behaviors. In the Dom-Inst test, subordinate subjects exhibited increased self-grooming and social contact during the aggressive encounter compared to the RI test, whereas dominant animals did not show these changes in the Sub-Inst test. Self-grooming is an innate behavior exhibited by various animal species and it is observed in both stressful and comfort conditions, and the interpretation of the change in self-grooming is condition dependent (Fentress 1977; Sachs 1988). Increase of self-grooming in the stressful condition is considered as displacement behavior to reduce arousal (Spruijt et al. 1992). Therefore, the increased self-grooming observed in subordinate subject may indicate a stress-related state caused by the pre-exposure to a dominant animal. On the other hand, during the instigator presentation, dominant males showed longer self-grooming when a subordinate opponent was presented as the instigator compared to the novel instigator. In contrast, subordinate males showed a slight, though non-significant, decrease in self-grooming when a dominant instigator was presented. Since self-grooming is also promoted when mice are in the relaxed condition as self-care behavior (Fentress 1977; Sachs 1988), grooming behavior during social instigation may reflect rather reduced stress. Taken together, the presentation of dominant and subordinate instigators elicited some distinct behavioral responses, suggesting that different mechanisms may underlie their suppressive effects on social instigation-heightened aggression. Further studies should further explore these potential differences.

In addition, which social cues from the instigator contribute to the pro-aggressive effect of social instigation remains to be elucidated. Mice use various sensory modalities in the social context, including tactile, acoustic, visual and olfactory information. Among these, olfactory cues play a particularly important role in the expression of aggressive behavior (Clancy et al. 1984; Denenberg et al. 1973). It has been reported that urine from subordinate males contains fewer aggression-promoting pheromones compared to urine from dominant males (Harvey et al. 1989). However, in our study, neither dominant nor subordinate instigators increased aggressive behavior in subsequent agonistic encounters. This suggests that the modulation of the pro-aggressive effect by a dominant-subordinate relationship may involve modalities other than olfactory cues. Once a dominant-subordinate relationship is established, animals often exhibited behavioral adaptations that deescalate aggression, as such submissive posturing by the subordinate (Ginsburg and Allee, 1942). Although we did not observe clear behavioral differences in the test animals during the social instigation procedure between novel instigators and dominant/subordinate instigators, it remains possible that subtle behavioral cues, undetected in the current study, may modulate the pro-aggressive effect of social instigation. The use of anesthetized instigators in future experiments would allow for the assessment of potential behavioral contributions to this phenomenon.

Sex difference in this phenomenon also need to be explored. Previous studies have shown that social instigation enhances aggressive behavior in female hamsters and postpartum rats (Da Veiga et al. 2011; Potegal and TenBrink 1984). The use of the female mouse rival aggression model (Newman et al. 2019), which shows aggression dynamics similar to those observed in males, will allow us to examine whether the social factors that modulate aggression are comparable between male and female mice.

## Conclusions

The social instigation procedure, in which exposure to a potential rival male instigator precedes a direct agonistic encounter, induces a clear pro-aggressive effect in male mice when the instigator is a novel male or a familiar male without a pre-established hierarchical relationship with the test animal. However, this effect is suppressed when the instigator has an already established dominant-subordinate relationship with the test animals. Our findings suggest that aggressive arousal induced by social instigation is influenced by the cognitive evaluation of social relationships, and social novelty combined with an ambiguous social hierarchy facilitates social instigation-heightened aggression.

## Data Availability

The datasets generated and analyzed during the current study are available from the corresponding author on request.
